# Hydration-stable PVA-based skin phantom for wearable biopotential sensor evaluation

**DOI:** 10.1038/s41598-026-49790-8

**Published:** 2026-04-28

**Authors:** Robert Lin, Max Gonzaga, Christopher L. Lewis, Krittika Goyal

**Affiliations:** Department of Mechanical and Mechatronics Engineering Technology, Rochester Institute of Technology, 1 Lomb Memorial Drive, Rochester, NY 14623 USA

**Keywords:** Skin phantom, Impedance, Wearable sensors, Hydration stability, Hydrophilic additives, Freeze-thaw cycles, Engineering, Materials science

## Abstract

The development of biomimetic skin phantoms is critical for reliable biopotential measurements in wearable sensor applications. A major limitation is hydration instability, as moisture loss alters ionic conductivity and leads to unpredictable impedance behavior. Building on prior work using polyvinyl alcohol (PVA) cryogels as benchtop analogs for electrode evaluation, this study investigates hydrophilic additive integration and freeze-thaw processing as complementary strategies to improve hydration retention and electrical stability. Seven commercial hydrophilic additives were screened for their ability to reduce mass loss and stabilize impedance over time. Aloe Vera proved most effective, reducing mass loss and impedance variability and extending phantom electrical lifespan from approximately 4 days for untreated controls to up to 18 days under optimized conditions. Embedded additive testing identified an optimal concentration of approximately 0.75 g (5.5-–6.5% w/w), while higher concentrations produced oversaturation and electrical drift. Freeze-thaw optimization demonstrated that a single cycle preserved structural integrity and minimized dehydration, whereas repeated cycling (≥ 2 cycles) increased mass loss, permittivity decay, and electrical instability. Differential scanning calorimetry quantified the distribution of freezable and bound water within the cryogel matrix. Samples containing the additive exhibited markedly reduced endothermic peak magnitudes during melting, indicating a lower fraction of freezable water and a corresponding increase in tightly bound water. These results establish a combined chemical-physical framework that extends phantom functional lifespan by more than fourfold and provides long-term hydration stability and consistent impedance behavior suitable for repeatable wearable biosensor testing.

**Supplementary Information:**

The online version contains supplementary material available at 10.1038/s41598-026-49790-8.

## Introduction

Biopotential measurements including electrocardiography (ECG), electromyography (EMG), and electroencephalography (EEG) are foundational to clinical diagnostics and emerging wearable health monitoring technologies^1–3^. The rapid adoption of long-term and wearable physiological monitoring systems has accelerated the development of novel electrode materials, geometries, and surface treatments aimed at improving comfort, signal fidelity, and durability^4^. However, biopotential recordings are highly sensitive to the electrode-skin interface, which varies significantly across individuals and environmental conditions^1–8^. Because skin impedance is strongly frequency and hydration-dependent, and at low frequencies is dominated by the stratum corneum, native skin cannot serve as a stable or reproducible testbed^5,6,8,9^.

This variability has motivated the development of synthetic skin phantoms with controlled and repeatable electrical and hydration properties^4–7^. Among candidate materials, polyvinyl alcohol cryogels (PVA-c) are widely used due to their mechanical compliance, tunable microstructure, and high water content^10–14^. However, conventional PVA-based skin phantoms suffer from progressive dehydration and time-dependent drift in electrical properties, limiting their functional lifetime and suitability for multi-day wearable sensor evaluation^5,9,15^. Existing models primarily replicate bulk conductivity and permittivity at moderate-to-high frequencies but do not accurately reproduce low-frequency electrode-skin interface impedance or maintain stable hydration over extended durations^16–18^. Consequently, current phantoms remain inadequate for standardized, long-term evaluation of wearable biopotential electrodes.

Several approaches have been explored to regulate phantom hydration, including controlled drying, immersion, humidity-controlled storage, and surface wetting techniques^9,15^. However, these methods offer limited precision, exhibit poor long-term stability, and fail to directly correlate hydration retention with electrical performance^5,9^. Thus, there remains a critical need for skin phantoms that provide both sustained hydration stability and reproducible low-frequency impedance characteristics.

**Fig. 1:**
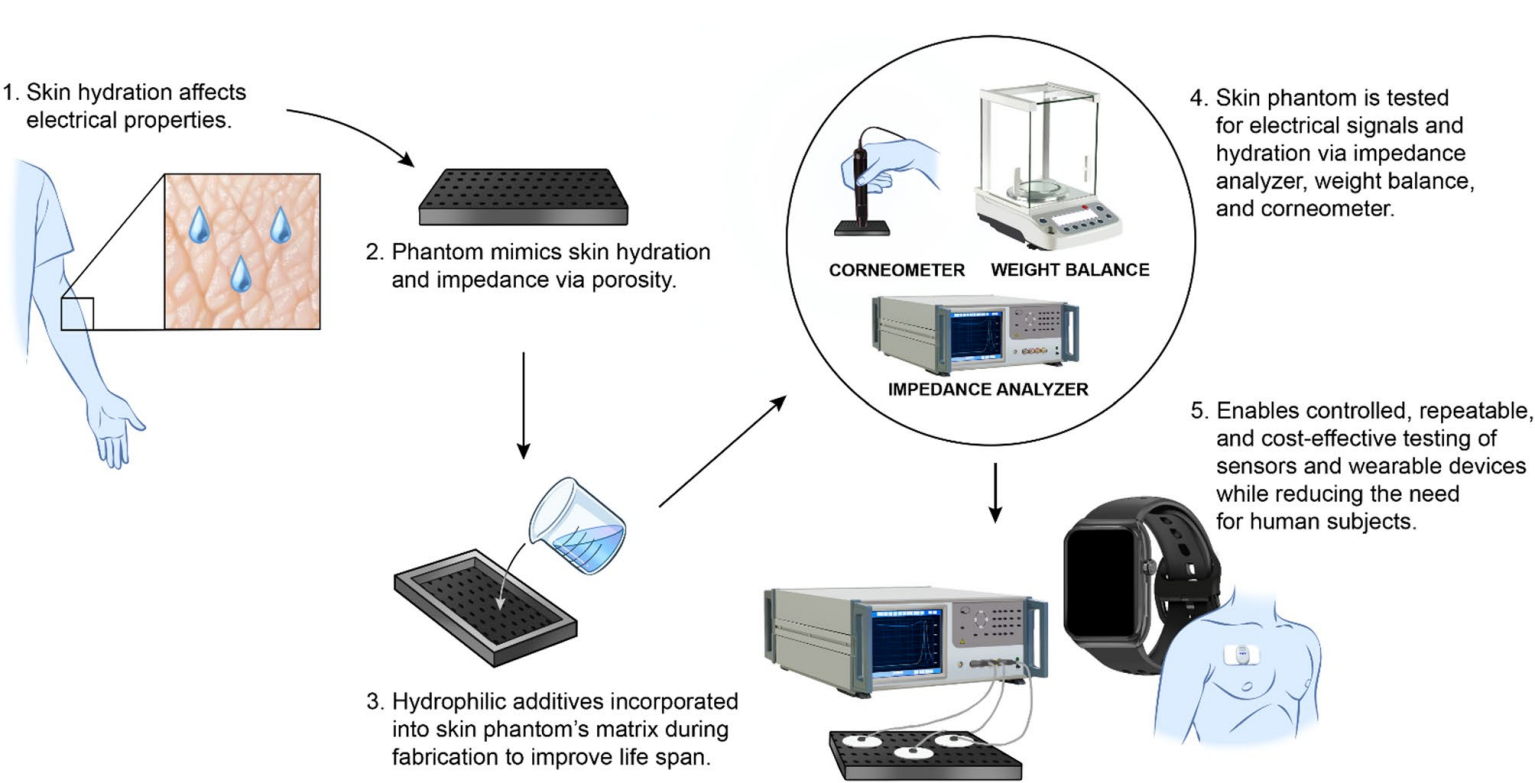
Overview of skin hydration mimicry, phantom fabrication, and electrical characterization. Clockwise from top-left: variation in human skin hydration and its effect on electrical properties; porous skin phantom designed to mimic hydration-dependent impedance; incorporation of hydrophilic additives into the phantom matrix during fabrication; hydration and electrical testing using a corneometer, weight balance, and impedance analyzer; application of the phantom for controlled, repeatable evaluation of wearable biosensors.

In this study, we address these limitations by systematically investigating complementary chemical and physical strategies to enhance hydration retention and low-frequency electrical stability in PVA-based skin phantoms. Hydrophilic additive incorporation and controlled freeze-thaw processing are evaluated in terms of mass loss, dielectric behavior, and impedance stability over time. Multiple commercial hydrophilic additives are screened, and differential scanning calorimetry is employed to quantify bound and free water fractions within the cryogel matrix, providing mechanistic insight into additive-driven hydration stabilization. This integrated thermal-electrical characterization framework establishes design principles for extending phantom functional lifetime and enabling reproducible, standardized validation of wearable biopotential sensors (shown in Fig. 1).

## Materials and methods

This study examines hydration stabilization in polyvinyl alcohol (PVA) cryogel skin phantoms for biopotential measurement applications. The primary objectives include (1) evaluating the effects of various hydrophilic additives on moisture retention and impedance consistency, and (2) assessing single and multi-cycle freeze-thaw processing for polymer network optimization.

### Skin phantom materials

The skin phantom was constructed using a two-layer material system designed to emulate the electrical and hydration-dependent behavior of human skin based on our previous work^5,6^. The upper layer, representing the stratum corneum, was based on polydimethylsiloxane (PDMS), a polymer with intrinsically high resistivity and low dielectric constant. Conductive carbon black and high-permittivity barium titanate (BaTiO₃) were selected as fillers to enable tuning of PDMS electrical properties toward physiologically relevant resistance and capacitance ranges.

The lower layer, representing hydrated dermal tissue, was composed of polyvinyl alcohol (PVA) cryogel. PVA was selected for its hydrophilicity, high water-retention capacity, and ability to form physically crosslinked networks through freeze-thaw cycling. When hydrated, PVA cryogels exhibit ionic conductivity and dielectric behavior similar to that of dermal tissue.

Together, these materials provide a platform capable of modeling both the resistive-capacitive behavior of the stratum corneum and the conductive, hydration-dependent properties of the underlying dermis. A photograph of the fully assembled two-layer phantom is provided in the Supplementary Information (Fig. S1). An additional image of the isolated lower-layer PVA cryogel used during additive screening and freeze-thaw testing is shown in Fig. S2 to illustrate the physical configuration of samples evaluated in those experiments.

### Skin phantom fabrication

**Fig. 2:**
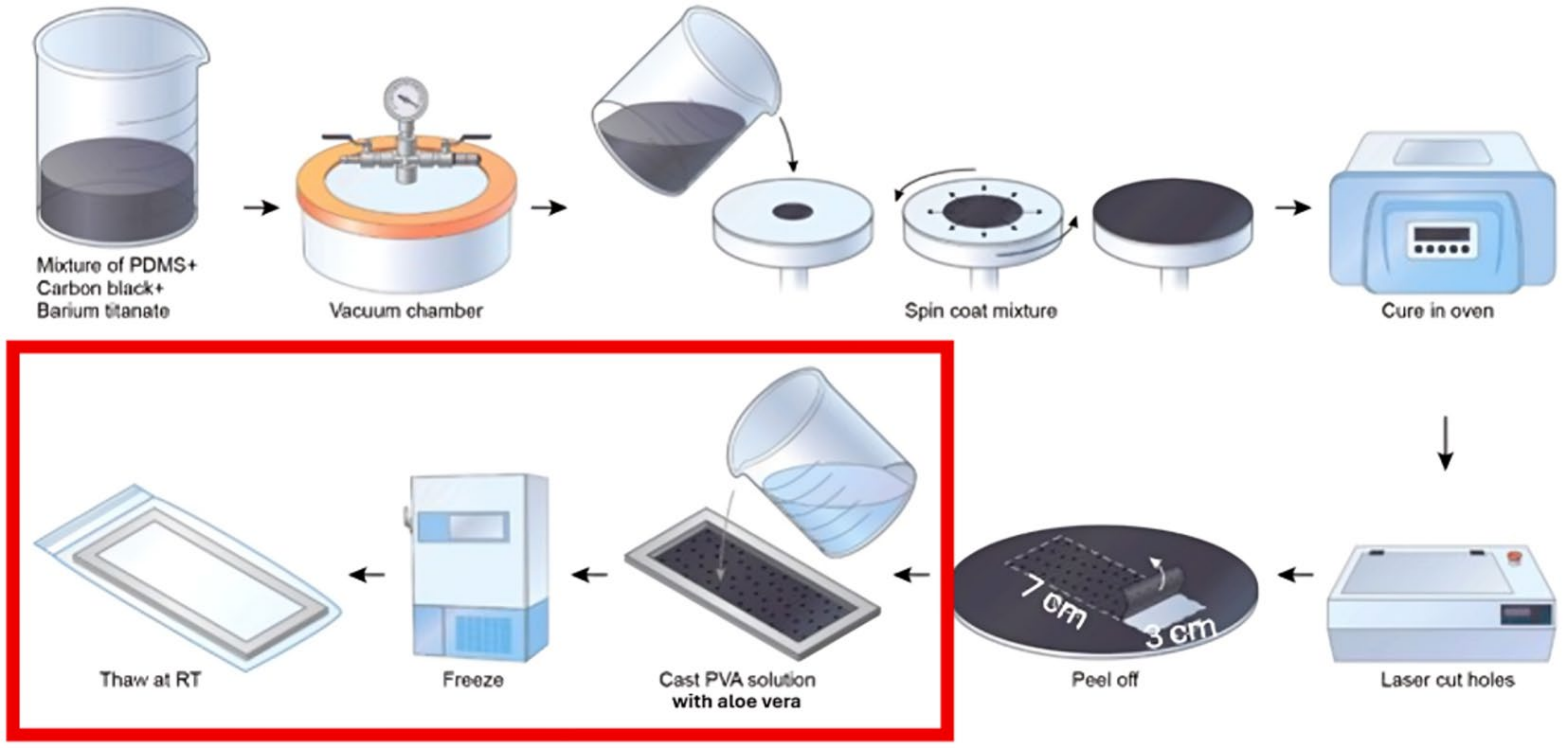
Steps for the fabrication process of a two-layered phantom. Clockwise from top-left: a mixture of PDMS, carbon black, and barium titanate; followed by removal of air bubbles in the vacuum chamber; spin coat mixture at 1000 RPM and 30 s; cure in an oven at 80 °C for two hours; laser-cut holes; peel off the layer; cast PVA solution; freeze (12 h at − 20 °C); thaw (24 h at RT) modified from^5^.

The fabrication process for the two-layer skin phantom followed a sequential workflow that integrates the materials described in Section “Skin phantom materials” into a unified structure. Figure 2 provides an overview of the complete fabrication pathway, including composite preparation, degassing, spin-coating, thermal curing, pore formation, cryogel casting, and freeze-thaw crosslinking. These steps establish the mechanical, dielectric, and hydration-dependent properties of each layer and ensure consistent layer-to-layer integration. Detailed procedures for fabricating the stratum corneum-mimicking PDMS layer and the PVA cryogel dermal layer are provided in Section “Stratum corneum layer fabrication” and “PVA cryogel layer fabrication”.

#### Stratum corneum layer fabrication

The upper layer consisted of a PDMS-based composite engineered to emulate the mechanical stiffness and dielectric behavior of the stratum corneum. PDMS was prepared at an increased curing ratio to enhance crosslinking and mechanical stability, enabling fabrication of an approximately 100 μm-thick film. To tailor electrical properties, the matrix was sequentially doped with high-permittivity barium titanate (BaTiO₃) to increase dielectric constant and conductive carbon black to reduce electrical resistance. The mixture was mechanically homogenized, degassed to remove entrapped air, spin-coated to achieve uniform thickness, and thermally cured. The PDMS composite formulation followed our prior work^5^, consisting of PDMS base and curing agent doped with 40% w/w barium titanate (BaTiO₃) and 2.5% w/w carbon black, which were previously shown to yield physiologically relevant resistive-capacitive behavior. No additional optimization of filler fractions was performed in the present study.

Laser cutting was subsequently used to introduce hydration-modulating pores and define a central rectangular geometry. Pore size and distribution were adjusted to tune porosity. Porosity refers to the proportion of void space within a material and, in this study, was quantified as the area fraction of open regions relative to the bulk material, given the uniform thickness of both pore structures and surrounding matrix. This relationship is formalized in Eq. (1) below.

1 $$\O = \frac{{No.\:of\:pores\: \times \:A_{{pore}} }}{{A_{{bulk\:material}} }}$$

where, $\:{A}_{pore}$ is the area of the pore, $\:{A}_{bulk\:material}$ is the area of bulk material, and the $\O$ represents porosity.

#### PVA cryogel layer fabrication

The lower layer of the skin phantom, representing hydrated dermal tissue, was fabricated using polyvinyl alcohol (PVA) cryogel. A 0.9% saline solution was prepared and heated to promote dissolution before 8.8 g of PVA powder was gradually incorporated under continuous stirring, corresponding to a 17.6% w/v PVA concentration, consistent with the formulation reported in^5^. The mixture was reheated as needed until a homogeneous, translucent gel formed, indicating complete polymer dissolution.

**Fig. 3:**
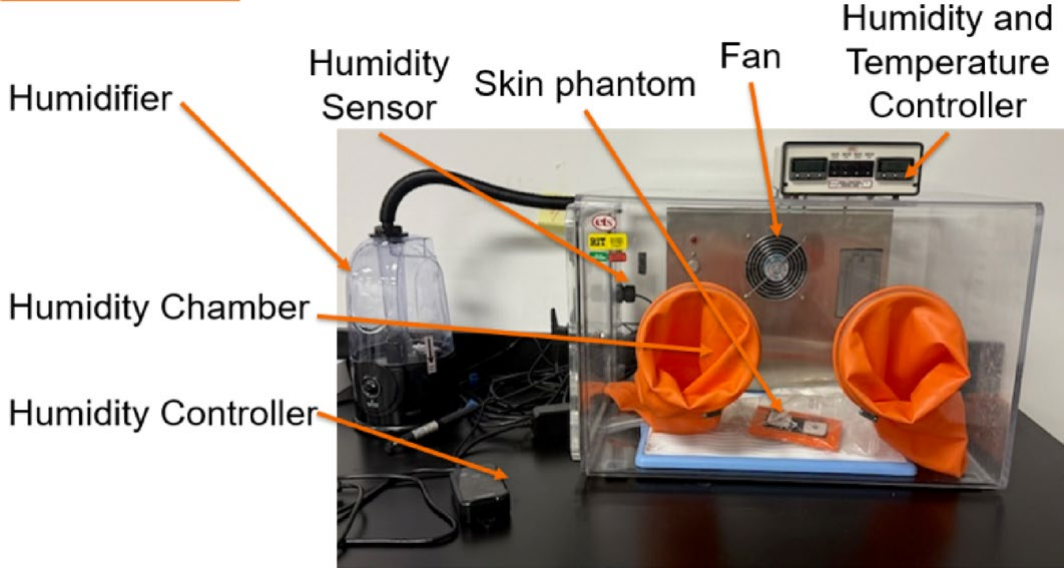
Controlled humidity chamber used for skin phantom thawing, storage, and hydration preservation. The system includes a humidifier, humidity and temperature controller, humidity sensor, circulation fan, and enclosed test chamber to maintain stable environmental conditions.

After cooling to room temperature and removal of surface bubbles, the solution was poured into a custom mold, with the upper layer positioned at the base when applicable. For preliminary characterization, the upper layer was excluded to enable isolated evaluation of the cryogel. The filled mold underwent a single freeze-thaw (FT) cycle, consisting of freezing at − 20 °C to initiate physical crosslinking, followed by controlled thawing at room temperature. Thawing was performed under high-humidity conditions within a sealed enclosure to minimize dehydration and promote moisture equilibration during polymer stabilization, as illustrated in Fig. 3.

### Measurement equipment

Hydration and electrical properties were characterized using three integrated instrumentation systems focused on electrical stability, moisture retention, and mass tracking. Impedance and phase-angle measurements were acquired using a Wayne Kerr 6500B Series Impedance Analyzer with dry EEG/EMG/ECG sensors (Neurotrode, Biomedical Instruments) and DIN snap leads^19^. A custom FDM-printed attachment (Prusa XL, Prusa) was integrated with a push-pull force gauge (Mxmoonfree) to maintain a consistent vertical pressure of approximately 300 Pa (2.25 mmHg), reducing placement variability and ensuring repeatable electrode contact.

Surface moisture levels were quantified using a Corneometer CM 825 (Courage & Khazaka Electronic GmbH, Cologne, Germany) connected to a Multi-Display Device (MDD4, MEDELINK, Brossard, QC, Canada). Measurements were recorded in arbitrary units (AU) at four locations surrounding the phantom center, enabling spatially consistent hydration assessment while minimizing edge effects and additive pooling. These four points (C1-C4) were averaged to determine hydration retention following treatment and thawing.

**Fig. 4:**
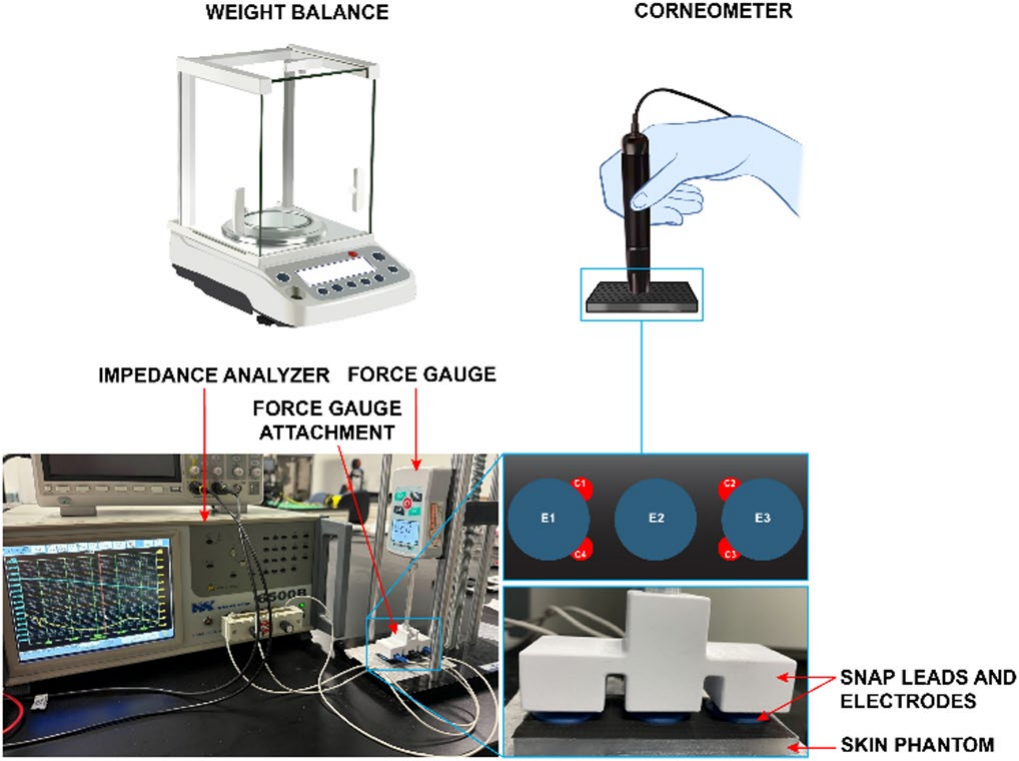
Experimental setup with analytical balance, corneometer, and impedance analyzer. Clockwise from top-left: an analytical weight balance used for mass measurements; a handheld corneometer probe resting on its baseplate for hydration measurements; an impedance analyzer and force gauge setup including a force gauge attachment with electrodes and snap leads connected.

Phantom mass was tracked using a PR224/E Analytical Balance (OHAUS, Parsippany, NJ, USA) to quantify water loss through evaporation and dehydration across additive and freeze-thaw conditions. Figure 4 illustrates the complete experimental setup, including the corneometer, analytical balance, and impedance analyzer with the force-gauge-mounted electrode assembly, as well as the measurement locations (C1-C4) and electrode positions (E1-E3) on the phantom surface.

### Application of hydrophilic additives

To investigate strategies for hydration enhancement, this study adopted a two-tiered approach to hydrophilic additive testing. The first phase involved topical application of additives on isolated lower-layer PVA cryogel phantoms to characterize post-thaw hydration behavior and electrical properties without interference from upper-layer structures. The second phase embedded the optimal additive directly into the phantom matrix during fabrication, enabling crosslinked integration and concentration-based evaluation within stratified skin phantom constructs. This framework allowed for comparative analysis of additive performance, balancing hydration retention, electrical consistency, and structural compatibility across experimental conditions.

#### Topical additive evaluation

The initial phase of hydration stability testing focused exclusively on isolated lower-layer PVA cryogel phantoms to evaluate the effects of hydrophilic additives without interference from upper-layer structures. Seven commercial additives, including trehalose (Trehalose Powder, Sugar Alternative & Sugar Substitute, 1 kg, 5 g per serving, BulkSupplements.com, Henderson, NV, USA), glycerin (Vegetable Glycerin, 100% Pure, Softening & Moisturizing, 4 oz, NOW Foods, Bloomingdale, IL, USA), hyaluronic acid (Hyaluronic Acid 2% + B5, Multi-Depth Hydration Serum, 30 mL, The Ordinary, Toronto, ON, Canada), propylene glycol (Propylene Glycol, 250 mL, Highest Purity, Manufactured in the USA, USP Food and Pharmaceutical Grade, Houston, TX, USA), urea (Urea Fertilizer 46.0% Minimum Nitrogen, Commercial Grade 99+% Purity, 0.9 lbs, OULLYY, Xiamen, Fujian, China), Aloe Vera (Aloe Vera Gel for Face, Skin, Hair & Sunburn Relief, 100% Pure, 300 mL, Miracle Lab Co., Ltd, Gwangmyeong-si, Gyeonggi-do, South Korea), and jojoba oil (USDA Organic Jojoba Oil, Cold-Pressed, 100% Pure & Natural, 8 fl. oz, Handcraft Blends, Austin, TX, USA), were applied topically post-thaw to enable controlled observation of treatment-induced hydration and electrical changes^20–30^. Each additive was tested across three phantom samples to support statistical consistency and assess intra-treatment variability. The without-additive control condition was tested using five independent phantom samples (*n* = 5) to establish a robust baseline for electrical variability. Variability is reported as the sample standard deviation (SD) for each condition.

For trehalose and urea, aqueous solutions at 10%, 20%, and 75% concentrations (by weight) were prepared using deionized water to also enable indirect assessment of increasing water concentrations, while other additives were applied in their native commercial forms. Following application until saturation, phantoms were allowed to stabilize overnight before instrumentation testing began. Hydration retention was evaluated using corneometer readings (in arbitrary units) and mass tracking, while electrical consistency was assessed by measuring impedance and phase angle variation across three sequential testing days.

Hydrophilic additives were incorporated at predefined weight fractions relative to the total phantom mass. The following concentrations were evaluated: jojoba oil (0.97%, 1.91%, 2.29% w/w), propylene glycol (0.86%, 3.10%, 8.64% w/w), trehalose (0.82%, 3.70%, 19.55% w/w), glycerin (0.41%, 0.82%, 2.50% w/w), hyaluronic acid (0.86%, 3.10%, 8.64% w/w), urea (0.72%, 3.94%, 4.36% w/w), and aloe vera (2.77%, 3.34%, 3.37% w/w). These concentrations were selected to span low, medium, and high additive loading levels and were not optimized beyond the ranges shown.

To accommodate additive stabilization timelines and avoid baseline discontinuities, measurements were collected across three consecutive days to evaluate topical hydrophilic treatments, rather than comparing post-treatment effects directly to Day 1 measurements. Impedance and phase angle fluctuations were analyzed across measurement days to characterize additive performance relative to without additive controls. This approach enabled quantification of each additive’s impact on hydration longevity and biopotential stability during early phantom lifespan. Because the study objective was comparative trend identification rather than hypothesis testing, no formal statistical significance tests (e.g., ANOVA, t-tests) were applied to the additive-screening datasets. Differences between treatments were interpreted based on consistent directional trends and variability (mean ± SD) rather than statistical significance.

#### Embedded Additive Integration

Following the initial screening of hydrophilic additives, Aloe Vera was selected for embedded-additive evaluation based on its performance in the hydration and electrical stability assessments described in Sections “Initial screening of hydrophilic additives for hydration and electrical stability” and “Quantitative comparison and additive selection”. This selection reflected a qualitative synthesis of predefined comparison criteria: relative mass loss, permittivity loss, and impedance and phase-angle stability, rather than a weighted or statistically optimized ranking. Aloe Vera was therefore incorporated directly into the lower-layer PVA cryogel matrix to determine whether embedded integration could further enhance phantom longevity and electrical reliability beyond surface application.

A concentration sweep ranging from 0.5 to 1.5 g of Aloe Vera, in 0.25 g increments, was performed by mixing the additive directly into the PVA solution prior to molding. This integration approach enabled crosslinking of the hydrophilic additive during the freeze-thaw polymerization process, promoting uniform dispersion and long-term hydration support. Embedded-additive electrical behavior was evaluated at a fixed porosity level, which was held constant to isolate concentration-dependent effects.

Electrical behavior was monitored across multiple days post-fabrication, with Day 1 serving as the baseline for impedance and phase-angle percent-change calculations across the frequency spectrum. Comparisons were made against without-additive and surface-applied phantoms to identify an embedded concentration that minimized conductivity fluctuation while maintaining mechanical integrity.

Because each embedded-additive concentration was evaluated using a single phantom at the tested porosity level, these experiments represent exploratory, single-sample measurements rather than statistically powered comparisons. Formal hypothesis testing and standard-deviation-based variability analysis were therefore not applicable. Porosity was treated as a controlled variable during the primary optimization, and additional porosity conditions were examined separately (Table S1) to assess how porosity modulates additive performance. Although replication was limited at the fixed porosity level, the concentration-dependent trends were consistent with the multi-porosity dataset in Table S1, strengthening confidence in the identified optimal concentration window. This embedded-additive approach provided a clear concentration-response relationship and demonstrated improved moisture retention and electrical stability within the 5.5–6.5% w/w range, as detailed in Section “Initial screening of hydrophilic additives for hydration and electrical stability”.

### Freeze-thaw cycle optimization

The next phase was conducted alongside the screening of hydrophilic additives using the lower layer and involved subjecting the lower layer to controlled freeze-thaw cycles to analyze their impact on structural integrity and hydration retention^31–33^. Freeze-thaw cycles were evaluated to determine optimal polymer structure refinement for maintaining hydration stability. Five phantoms were fabricated and subjected to 1–5 freeze-thaw cycles, with each phantom assigned to a final cycle count. Because freeze-thaw processing is cumulative and irreversible, phantoms undergoing a higher number of cycles inherently pass through all preceding cycle states. This structure yielded five independent observations for the 1-cycle condition, four for 2 cycles, three for 3 cycles, two for 4 cycles, and one for 5 cycles. This progressive-treatment design enabled evaluation of cycle-dependent degradation while maintaining independent replication at each intermediate cycle count. Samples were frozen at − 20 °C for 12 h, followed by thawing at room temperature for 24 h in a sequential cycle like the fabrication process of the skin phantom; the number of cycles varied from 1 to 5 to simulate the skin phantom’s expected usage period, as the skin phantom is typically tested over a 5-day timeframe, allowing comparison between different conditions^5,31–33^. Hydration loss alongside impedance and phase changes were measured after each cycle to determine optimal processing conditions for improving durability. Because the study objective was comparative trend identification rather than hypothesis testing, no formal statistical significance tests (e.g., ANOVA, t-tests) were applied to the freeze-thaw datasets. Differences between treatments were interpreted based on consistent directional trends and variability (mean ± SD) rather than statistical significance.

### Impedance and phase angle range metrics

For each sample, the impedance range ($\:{Z}_{range}$) and phase‑angle range ($\:{\theta\:}_{range}$) were computed as the difference between the maximum and minimum values across the 20 Hz–5 MHz sweep. The reported values represent the arithmetic mean of these per‑sample ranges across replicates. This metric therefore reflects the average impedance range and average phase‑angle range for each treatment condition. This approach therefore reflects the average impedance range and average phase-angle range for each condition and provides a concise descriptor of broadband electrical variability.

### Differential scanning calorimetry

Once the optimal concentration of additive was determined, a sample of about 0.5 mg was then extracted from the skin phantom and was placed into a Differential Scanning Calorimeter (DSC 250, TA Instruments, New Castle, DE, USA), where it was tested under the following parameters: (1) equilibrated at 40.00 °C, (2) ramped 1.00 °C/min to − 40.00 °C, held at − 40.00 °C for 5 min, and then ramped 2.00 °C/min to 40.00 °C^34^.

### Electrical stability and failure criterion

Electrical death was defined using a logarithmic thresholding method (Eq. 2), in which a deviation of one order of magnitude (10×) from the Day 1 baseline impedance was used as the failure criterion. This threshold was motivated by prior work indicating that order-of-magnitude impedance shifts fall outside the physiologically realistic range of human skin impedance behavior^5^. Preliminary validation measurements in the present study confirmed that this threshold corresponded to the onset of functional instability in the phantom. Accordingly, the ± 1 log_10_criterion was established prior to full data collection as the operational definition of electrical failure. This failure metric was applied specifically to the embedded-additive experiments, where long-term electrical lifespan was evaluated relative to Day 1. Additive-screening and freeze-thaw analyses used impedance-range and hydration-loss metrics rather than the electrical-death threshold.

Impedance and phase angle measurements were collected across 20 Hz–5 MHz, corresponding to the operational bandwidth of the Wayne Kerr 6500B analyzer. The lower bound of 20 Hz reflects the minimum measurable frequency of the instrument; although physiological biopotentials such as EEG and ECG contain components below 20 Hz, these frequencies were not accessible due to equipment limitations. The upper bound of 5 MHz was retained to capture high-frequency dielectric behavior relevant for comparison with published human-skin impedance spectra and for future benchmarking applications^35^.

Biopotential-relevant frequency bands used for the log-deviation failure criterion (20–100 Hz for EEG/ECG and 20–500 Hz for EMG) were selected to fall within the measurable range while still encompassing the dominant spectral content of these signals. These ranges are consistent with prior literature on biopotential recording bandwidths^36,37^. A log_10_ deviation of ± 1 therefore served as a clear and interpretable marker of electrical failure. Across all tests, higher frequencies exhibited smaller log-scale shifts, indicating that degradation was more pronounced in the lower-frequency bands where stable impedance is most critical for signal fidelity.

2 $$\:\varDelta\:log={\mathrm{log}}_{10}\frac{Day\:\#}{Day\:1}\:$$

This framework enabled consistent identification of degradation onset in the embedded-additive phantoms and facilitated direct comparison between additive-treated and control samples.

## Results

This section presents experimental findings in a staged progression that reflects the sequence of analyses performed in this study. Section “Initial screening of hydrophilic additives for hydration and electrical stability” reports the results of hydrophilic additive screening, focusing on hydration retention and electrical stability in the lower‑layer PVA cryogel. Section “Quantitative comparison and additive selection” introduces the quantitative comparison framework and the explicit decision criteria used to identify the most suitable additive. Section “Electrical stability and lifespan modulation via embedded additive-porosity interactions” evaluates the performance of the selected additive when embedded within the phantom matrix, and Section “Impact of multiple freeze-thaw cycles” examines the effects of freeze-thaw processing on hydration and electrical behavior. To maintain clarity between empirical observations and interpretive conclusions, descriptive results are presented first within each subsection, followed by a brief interpretive summary at the end.

This study investigates how hydrophilic additives and freeze-thaw cycling influence hydration stability and electrical lifespan in PVA‑based skin phantoms. The upper‑layer stratum corneum simulation was intentionally excluded during the initial screening phase to isolate the behavior of the bulk gel matrix and evaluate additive performance without external protective effects. Additives were compared based on their ability to minimize mass loss and maintain consistent electrical behavior across repeated measurements. Rather than relying solely on absolute impedance or phase angle values, stability was assessed using the spread of impedance and phase angle across measurement days and across biopotential‑relevant frequency bands.

For each lower‑layer sample, impedance magnitude and phase angle were measured across the full 20 Hz–5 MHz frequency sweep. Electrical stability was quantified using the impedance range and phase angle range, defined as the difference between the maximum and minimum values across the sweep. These per‑sample ranges were averaged across three replicates for each additive condition to obtain the reported values in Figs. 6 and 10. No normalization was applied prior to averaging. This range‑based scalar metric provides a concise measure of broadband electrical variability that is directly relevant to biopotential sensor performance and is distinct from the log‑deviation metric used to evaluate electrical failure.

Because the impedance and phase‑angle range metrics collapse the full 20 Hz–5 MHz spectrum into a single scalar value, they capture overall spectral dispersion rather than frequency‑specific behavior. Different physical mechanisms dominate at low, mid, and high frequencies, and these effects cannot be separated within a broadband range metric. This limitation is most evident in conditions that exhibit strong frequency‑dependent behavior, such as the Aloe Vera treatment in Fig. 6B, where larger phase‑angle variability reflects underlying dispersion. Accordingly, the range‑based metric should be interpreted as a global descriptor of electrical stability across treatments, rather than a surrogate for behavior within specific biopotential frequency bands.

### Initial screening of hydrophilic additives for hydration and electrical stability

**Fig. 5:**
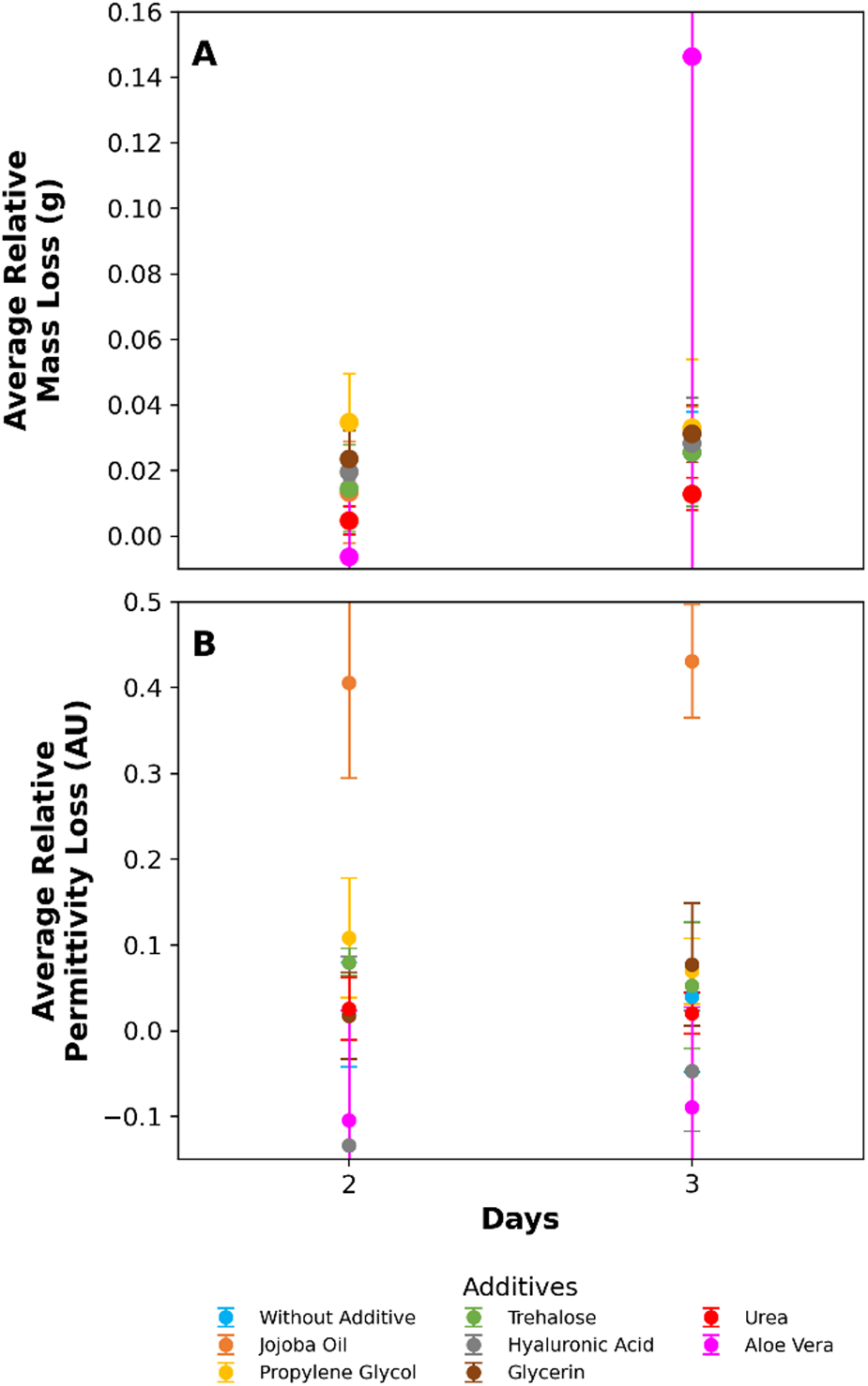
Comparison of hydrophilic additives on phantom stability over time. (**A**) average relative mass loss and (**B**) average relative permittivity loss measured at days 2 and 3 using day 1 as initial for phantoms fabricated with different additives. Error bars represent standard deviation. The without-additive condition reflects *n* = 5, while all additive conditions reflect *n* = 3.

Hydrophilic additives produced distinct effects on hydration retention and electrical stability within the lower-layer PVA cryogel samples. Because the without-additive control was evaluated with higher replication (*n* = 5), its variability estimate is more robust than those of the additive conditions (*n* = 3). Figure 5A shows the three-sample average relative mass loss for each treatment. Aloe Vera and urea exhibited reduced mass loss after stabilization on Day 2, indicating improved short-term hydration retention compared to the without-additive control. Other additives showed greater or increasing mass loss after Day 2, suggesting limited moisture-binding capacity. Relative mass and permittivity losses were calculated using Eq. (3), where *Initial* is the initial or baseline day of measurement, *Measured* is the day of comparison, and *Relative Loss* is the value loss relative to the initial day.

3 $$\:Relative\:Loss=\frac{Initial-Measured}{Initial}$$

Figure 5B presents the corresponding relative permittivity loss. Aloe Vera and hyaluronic acid showed reduced permittivity loss, indicating improved dielectric preservation. Other additives exhibited higher permittivity loss, reflecting reduced ability to maintain moisture-dependent dielectric properties.

**Fig. 6:**
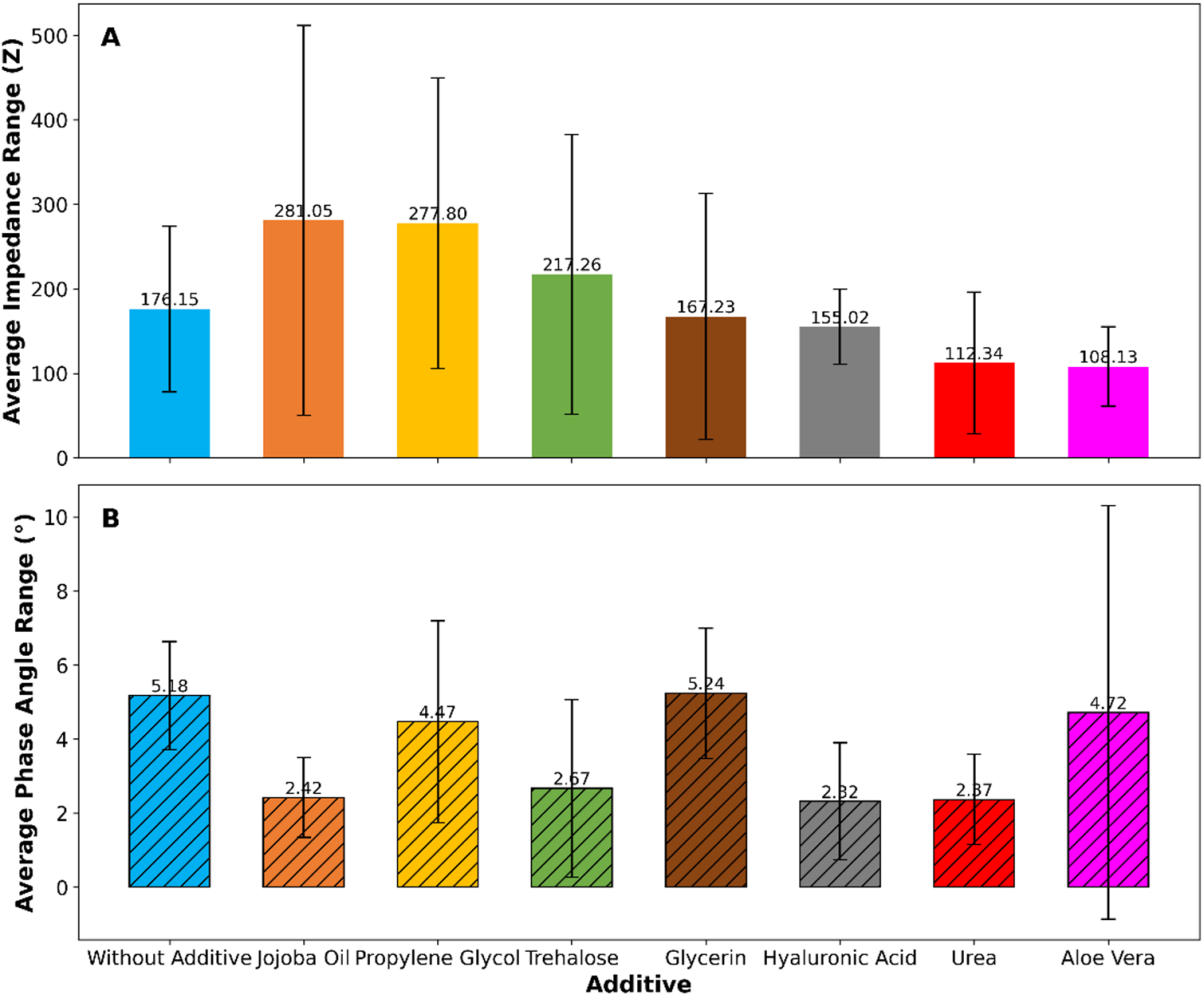
Electrical comparison of lower-layer hydrophilic additives. (**A**) Impedance range (Z) (averaged across replicates) for each additive, including the without-additive control (176.1 Ω), jojoba oil (281.05 Ω), propylene glycol (277.80 Ω), trehalose (217.26 Ω), glycerin (167.23 Ω), hyaluronic acid (155.02 Ω), urea (112.34 Ω), and Aloe Vera (108.13 Ω). (**B**) Phase angle range (θ) (averaged across replicates) for the same additives, ranging from 2.32° to 5.24°. Error bars represent standard deviation. The without-additive condition reflects *n* = 5, while all additive conditions reflect *n* = 3.

Jojoba oil (0.97%, 1.91%, and 2.29% w/w) produced elevated mass loss, high permittivity loss, and the largest impedance ranges (Fig. 6A), indicating substantial conductivity variability. Propylene glycol (0.86%, 3.10%, and 8.64% w/w) showed the highest mass loss and second-highest permittivity loss, accompanied by large impedance fluctuations and elevated phase-angle variation. Trehalose (0.82%, 3.70%, and 19.55% w/w) exhibited slightly greater mass loss than the control and elevated permittivity loss, with intermediate impedance stability. Glycerin (0.41%, 0.82%, and 2.50% w/w) resulted in higher mass loss and inconsistent permittivity retention, with elevated impedance and phase-angle variability as shown in both Fig. 6A,B. Hyaluronic acid (0.86%, 3.10%, and 8.64% w/w) produced greater mass loss than the control but showed low permittivity loss; however, impedance and phase-angle ranges remained large. Urea (0.72%, 3.94%, and 4.36% w/w) demonstrated reduced mass loss, lower permittivity loss, and smaller impedance and phase-angle ranges relative to most additives. Aloe Vera (2.77%, 3.34%, and 3.37% w/w) showed low mass loss on Day 2, reduced permittivity loss, and impedance and phase-angle ranges comparable to urea and hyaluronic acid. These comparisons are based on qualitative trends in mean behavior and variability (mean ± SD); no statistically significant differences between additives were tested or claimed.

Across all additives, urea, hyaluronic acid, and Aloe Vera produced the smallest conductivity fluctuations (Fig. S3). Trehalose and glycerin provided partial improvements, while propylene glycol and jojoba oil showed the least favorable hydration and electrical characteristics. Because the impedance and phase-angle extrema consistently occurred within the lower portion of the spectrum (approximately 20–1000 Hz, as shown in Fig. S3), the broadband range metric was effectively determined by the same low-frequency behavior that dominates biopotential interfaces. Examination of the EEG/ECG (20–100 Hz) and EMG (20–500 Hz) bands confirmed that the largest and smallest values within the full 20 Hz–5 MHz sweep originated from these lower bands. As a result, the broadband impedance and phase-angle ranges were numerically equivalent to the ranges computed within the application-relevant bands, and the relative ranking of additives did not change. Because the low-frequency behavior already governs the broadband metric, separate band-limited plots were not included to avoid redundancy.

The screening results show that several additives improved hydration retention and electrical stability relative to the control. Urea, hyaluronic acid, and Aloe Vera consistently reduced mass loss, permittivity loss, and impedance variability, indicating that these additives warrant further quantitative comparison. Section “Quantitative comparison and additive selection” applies predefined decision criteria to determine which additive provides the most balanced performance for subsequent embedded-additive evaluation.

### Quantitative comparison and additive selection

A quantitative decision framework was applied to identify the most suitable hydrophilic additive for long‑term hydration and electrical stability. Additives were evaluated using three predefined criteria: (1) minimizing relative mass loss, (2) minimizing permittivity loss, and (3) minimizing impedance and phase‑angle range across the 20 Hz–5 MHz spectrum. Additives that simultaneously reduced hydration loss and electrical variability were considered superior. These criteria were applied qualitatively rather than through a weighted or statistical optimization, and the selection reflects a synthesis of observed trends rather than a predefined or statistically ranked scoring system.

Using this framework, urea, hyaluronic acid, and Aloe Vera emerged as the strongest candidates based on their performance in Section “Initial screening of hydrophilic additives for hydration and electrical stability”. Urea demonstrated low mass loss and stable electrical behavior but exhibited structural softening at higher hydration levels. Hyaluronic acid preserved dielectric properties but showed larger impedance and phase‑angle fluctuations. Aloe Vera provided low Day 2 mass loss, the lowest permittivity loss across both days, and stable impedance and phase‑angle ranges without compromising structural integrity.

Although Aloe Vera exhibited the highest mass loss on Day 3, hydration stability cannot be evaluated using mass loss alone. Permittivity loss provides a complementary measure of bound‑water retention, and Aloe Vera showed lower permittivity loss across both days compared to urea and hyaluronic acid. When considered together with electrical stability, Aloe Vera was the only additive that performed consistently well across all predefined criteria. Urea and hyaluronic acid demonstrated slightly lower electrical variability but did not provide comparable hydration stability. For this reason, Aloe Vera was selected based on its combined performance rather than any single metric.

Based on the combined hydration and electrical stability metrics and the predefined decision criteria, Aloe Vera was identified as the most suitable additive for further evaluation. This designation guided the embedded-additive experiments presented in Section “Electrical stability and lifespan modulation via embedded additive-porosity interactions”, where Aloe Vera was incorporated directly into the phantom matrix to assess its performance under extended drying and electrical stress conditions.

### Electrical stability and lifespan modulation via embedded additive-porosity interactions

**Fig. 7:**
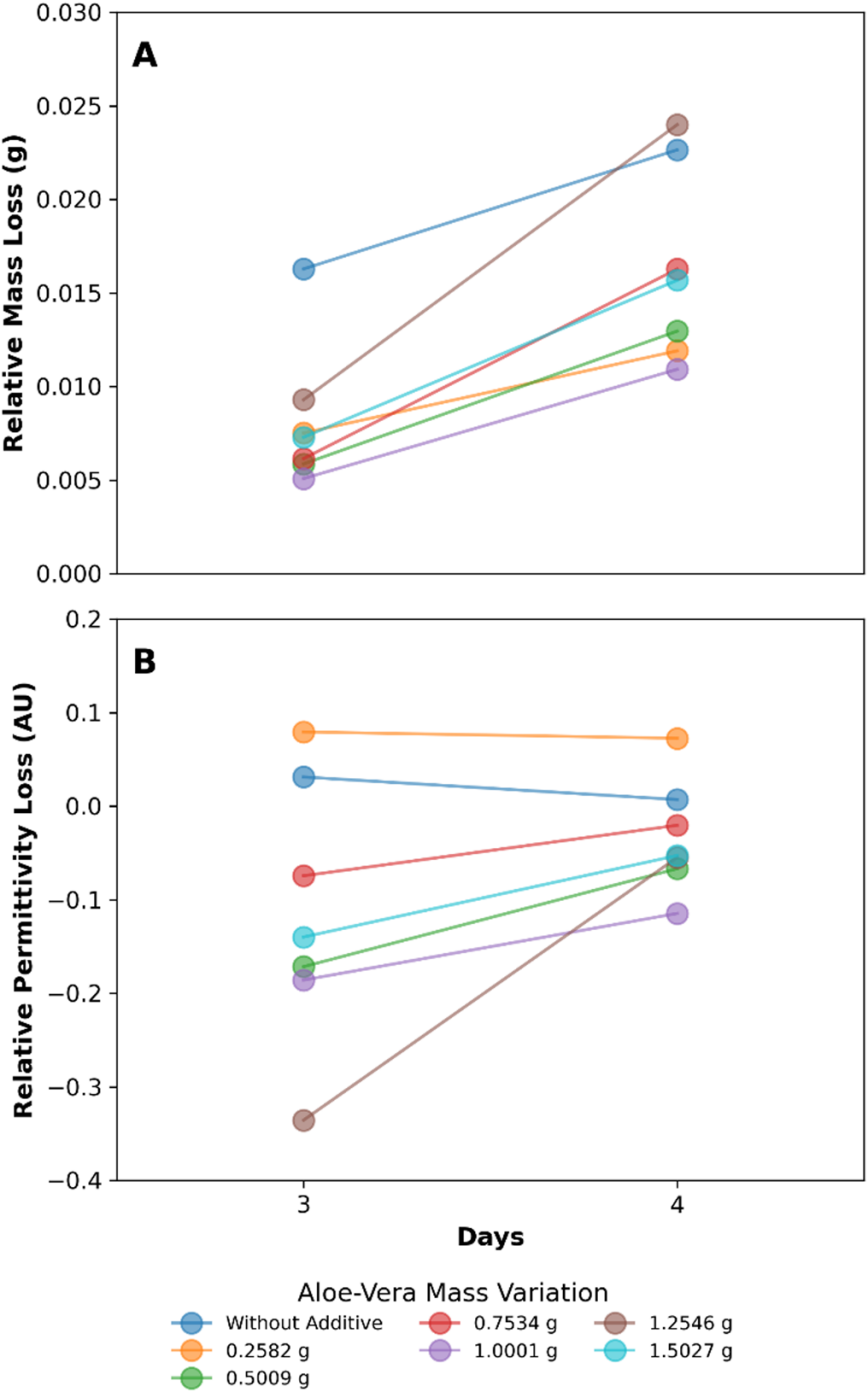
Varying concentration (**A**) relative mass loss and (**B**) relative average permittivity loss over time. Panel A depicts the relative mass loss (g) from day 3 to day 4 for various initial masses of aloe-vera and a control (without additive); all samples show increased mass loss on day 4 compared to day 3. Panel B depicts the relative permittivity loss (AU) over the same day 3 to day 4 period, showing similar trends to mass loss, where all samples exhibit greater permittivity loss on day 4 compared to day 3.

Following the identification of Aloe Vera as the most suitable hydrophilic additive in Section “Quantitative comparison and additive selection”, embedded-additive formulations were evaluated to determine how Aloe Vera concentration and phantom porosity influence hydration retention, electrical stability, and overall lifespan. Embedded treatments were incorporated directly into the lower-layer phantom matrix to assess their performance under ambient drying conditions relative to additive-free controls. Relative mass and permittivity losses were calculated using Eq. (3), and log-scale impedance and phase-angle shifts were assessed using the thresholding method described in Eq. 2. The concentration-dependent trends shown in Fig. 7 were evaluated at a single porosity level, while Table S1 provides additional porosity conditions that illustrate how porosity and additive concentration interact to influence electrical lifespan.

Figure 7 shows the hydration behavior of phantoms containing varying concentrations of Aloe Vera at 1.25% porosity. Relative mass loss decreased with increasing Aloe Vera content, with the 1.0 g formulation exhibiting the lowest mass loss between Days 3 and 4. Relative permittivity loss showed similar values across concentrations, although all embedded-additive samples exhibited lower permittivity loss than the without-additive control.

Electrical behavior further demonstrated the influence of embedded Aloe Vera concentration. Additive-free phantoms showed rapid degradation, with substantial impedance and phase-angle shifts by Day 4. In contrast, phantoms embedded with 6.70% w/w Aloe Vera maintained stable impedance and phase-angle profiles across the full frequency range through Day 15 and beyond. Additional samples with concentrations between 5.5 and 6.5% w/w exhibited similar stability trends (Table S1), indicating consistent performance across this concentration range.

A clear phase-angle relaxation was observed in the impedance spectra, with the relaxation frequency shifting as dehydration progressed, indicating a characteristic relaxation process within the bilayer phantom. While quantitative extraction of this behavior, such as computing dielectric loss or identifying the characteristic relaxation frequency, could provide additional insight into hydration-dependent changes in ionic mobility, interfacial polarization, or effective RC time constants, such electrical modeling falls outside the scope of the present work. The focus of this study was to evaluate hydration-stabilizing strategies rather than to develop a full dielectric relaxation model. Future work incorporating dielectric-loss analysis or relaxation-frequency tracking may therefore offer a more detailed mechanistic interpretation of the electrical evolution of the phantom system.

**Fig. 8:**
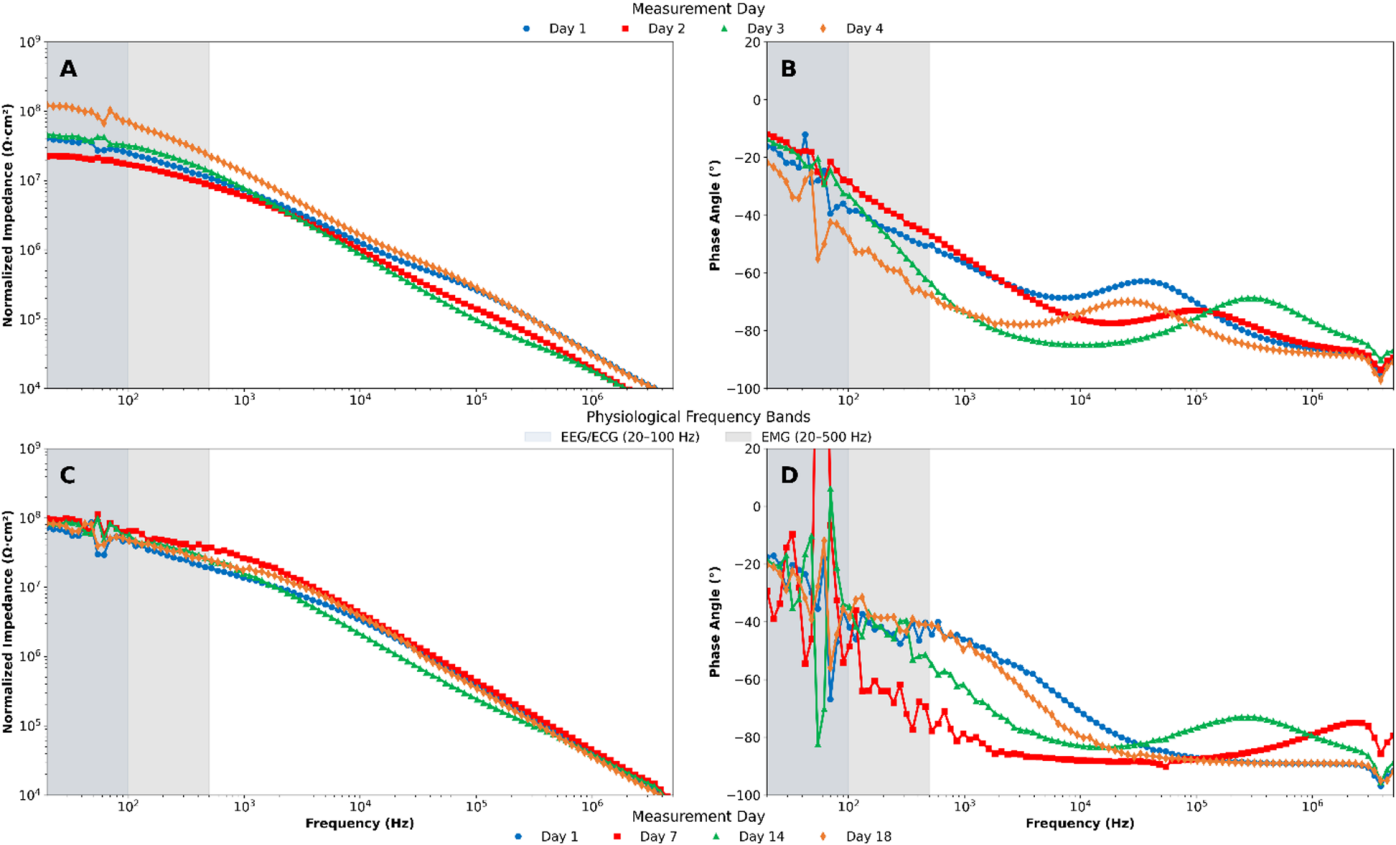
Without additive (**A**) normalized impedance and (**B**) phase angle over time vs. with additive 6.70% w/w (**C**) normalized impedance and (**D**) phase angle over time. Top left panel A shows the normalized impedance (Ω*cm^2^) versus frequency (Hz) for the without additive sample across day 1, day 2, day 3, and day 4, demonstrating a consistent decrease in impedance magnitude over time; followed by top right panel B which depicts the corresponding phase angle (°) versus frequency, exhibiting a pronounced shift in the phase minimum over the four days; followed by bottom left panel C which shows the normalized impedance versus frequency for the with additive sample across day 1, day 7, day 14, and day 18, demonstrating greater stability (less change over a longer period) compared to panel A; and finally bottom right panel D depicting the corresponding phase angle versus frequency, which also shows more consistent behavior over the 18-day period than panel B. Shaded regions denote physiologically relevant EEG/ECG (20–100 Hz) and EMG (20–500 Hz) frequency bands.

#### Influence of porosity on additive performance

To clarify the interaction between porosity and additive concentration, additional porosity conditions from Table S1 were examined. At the optimal concentration (0.75 g), lower-porosity phantoms exhibited the longest electrical lifespans, while higher-porosity samples showed reduced stability despite identical additive loading. This indicates that porosity modulates the effectiveness of Aloe Vera by altering moisture retention and ionic mobility within the cryogel matrix. Accordingly, the 5.5–6.5% w/w concentration window should be interpreted within the context of this porosity-dependent behavior. Although no statistical hypothesis testing was performed, the 0.75 g condition consistently produced the longest electrical lifespan across porosity levels, while lower concentrations performed well only at low porosity and higher concentrations exhibited diminishing returns and oversaturation-related instability. This cross-porosity robustness supports the designation of 0.75 g as the optimal concentration window. Concentrations above this range begin to invert the stabilizing effect, producing larger log-shift deviations and reduced long-term performance.

### Impact of multiple freeze-thaw cycles

**Fig. 9:**
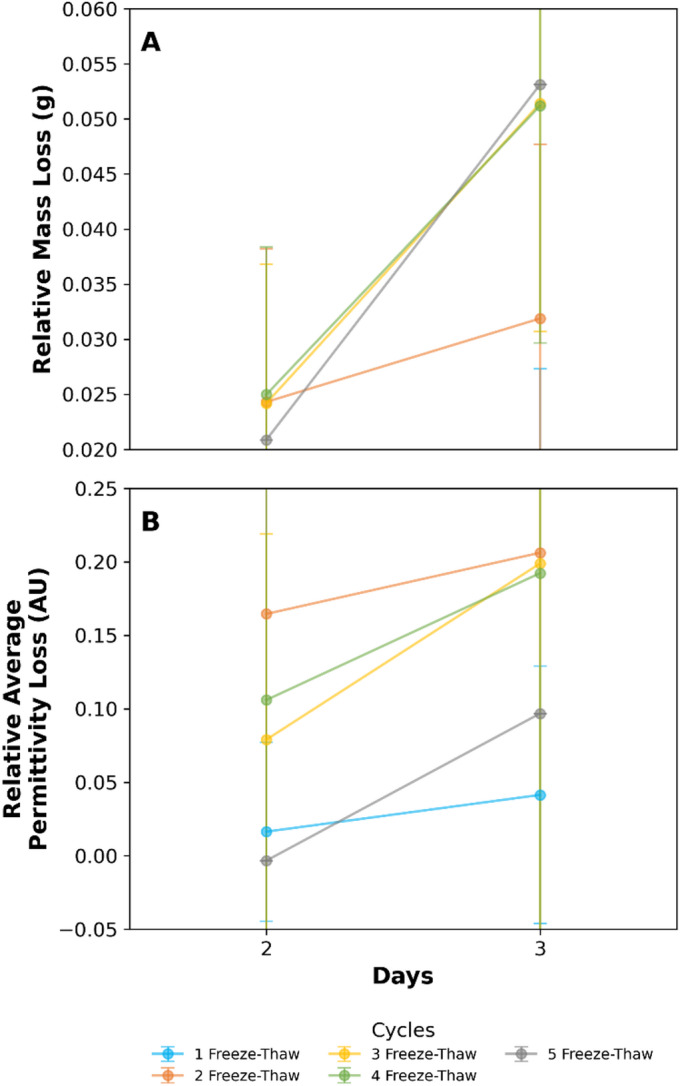
Comparison of freeze-thaw cycles on phantom stability over time. (**A**) average relative mass loss and (**B**) average relative permittivity loss measured at days 2 and 3 using day 1 as initial for phantoms fabricated with different freeze-thaw cycles. Error bars represent standard deviation for cycle counts with *n* > 1. The 5-cycle condition reflects a single sample.

Multiple freeze-thaw cycles were evaluated to determine their effects on hydration stability and electrical consistency in PVA skin phantoms. Because phantoms assigned to higher freeze-thaw counts necessarily pass through all earlier cycles, the dataset includes multiple independent observations for the lower cycle conditions (five for 1 cycle, four for 2 cycles, etc.). This nested structure allowed validation of cycle-dependent trends despite the cumulative nature of the treatment. Figure 9A shows that phantoms subjected to additional freeze-thaw cycles exhibited progressively greater relative mass loss between Days 2 and 3. Figure 9B shows a corresponding increase in relative permittivity loss with increasing cycle count. Relative mass and permittivity losses were calculated using Eq. (3).

**Fig. 10:**
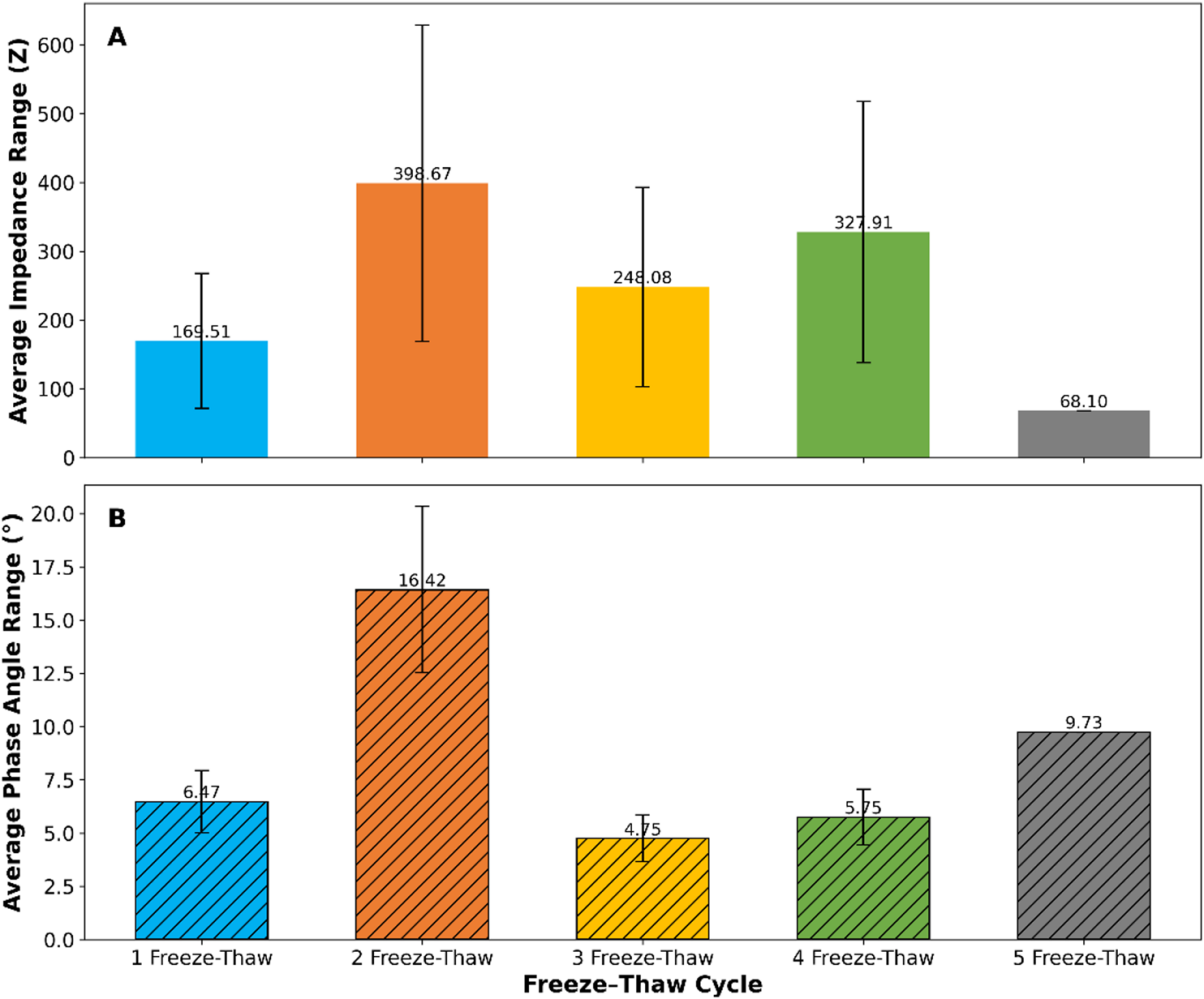
Electrical response of the lower layer across repeated freeze-thaw cycles. (**A**) Impedance range (Z) (averaged across replicates) for cycles 1–5, showing changes in electrical behavior with increasing freeze-thaw exposure. (**B**) Phase-angle range (θ) (averaged across replicates) for the same cycles, illustrating corresponding shifts in dielectric response. Error bars represent standard deviation for cycle counts with more than one sample (*n* = 5 for cycle 1, *n* = 4 for cycle 2, *n* = 3 for cycle 3, and *n* = 2 for cycle 4). The 5-cycle condition reflects a single sample (*n* = 1) and therefore has no error bars.

Impedance and phase-angle stability across freeze-thaw conditions are shown in Fig. 10A,B. Phantoms processed with two or more cycles displayed larger impedance ranges and greater phase-angle variability than the single-cycle control. The five-cycle condition exhibited the largest fluctuations, indicating substantial electrical instability. Comparisons between additive-free and additive-containing samples showed that the destabilizing effect of repeated cycling occurred regardless of additive presence.

The freeze-thaw results demonstrate that increasing cycle count accelerates dehydration, increases permittivity loss, and introduces substantial electrical variability. These trends indicate that repeated cycling disrupts the PVA network rather than strengthening it, leading to reduced structural integrity and compromised electrical reliability. Limiting processing to a single freeze-thaw cycle provides sufficient polymer crosslinking while preventing excessive softening and moisture loss, making it the most effective strategy for maintaining hydration retention and stable impedance behavior in long-term biosensor testing^31–33^. These electrical and hydration findings were consistent with preliminary visual observations (Fig. S4), which showed that a single freeze-thaw cycle produced a stable, water-retaining phantom, whereas room-temperature curing yielded a transparent thermoplastic, and multiple cycles led to water leakage and loss of integrity.

### Identifying the optimal skin phantom

The combined hydration and electrical stability results establish a multi-parameter framework for optimizing PVA-based skin phantoms capable of maintaining moisture and electrical fidelity over extended testing periods. Embedded additive type, additive concentration, and freeze-thaw processing were found to interact strongly, collectively determining phantom longevity, ionic conductivity, and structural robustness under dehydrating conditions.

Aloe Vera demonstrated the most favorable balance of hydration retention and electrical stability among the hydrophilic additives evaluated. Embedded concentrations near 0.75 g (5.5–6.5% w/w) minimized mass loss, reduced permittivity degradation, and produced the smallest impedance and phase-angle drift across the 20 Hz–5 MHz spectrum. Concentrations below this range (0.25–0.5 g) provided insufficient hydration stabilization, while higher concentrations (≥ 1.25 g) led to oversaturation, matrix softening, and erratic electrical behavior. These trends define a soft saturation threshold between 0.75 g and 1.0 g, beyond which electrical performance diverges from the stabilized baseline.

Freeze-thaw processing further influenced phantom performance. A single freeze-thaw cycle provided sufficient polymer crosslinking to support additive integration while preserving matrix integrity. Additional cycles (≥ 2) increased mass loss, accelerated dehydration, softened the gel structure, and amplified electrical instability, indicating that repeated cycling degrades rather than strengthens the PVA network.

Taken together, these findings define a clear optimal configuration for long-term hydration and electrical stability: approximately 0.75 g Aloe Vera embedded within the PVA matrix combined with a single freeze-thaw cycle. This formulation provides a reproducible and scalable foundation for durable, electrically consistent skin phantoms suitable for wearable biosensor validation.

### Differential scanning calorimetry (DSC) analysis

**Fig. 11:**
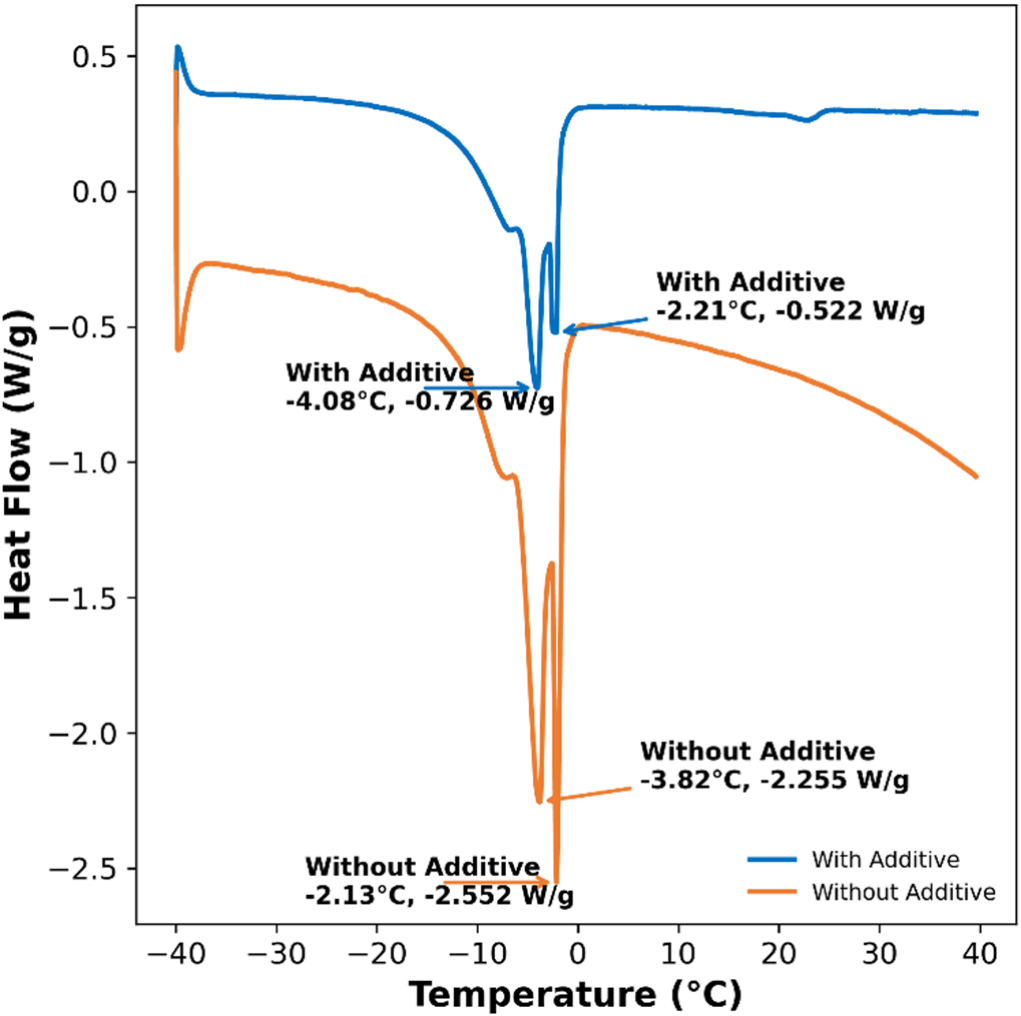
Differential scanning calorimetry of phantoms without additive and with additive. Without additive phantom shows two endothermic peaks: a primary peak at − 3.82 °C with a heat-flow magnitude of − 2.26 W/g, and a secondary peak at − 2.13 °C with a heat-flow magnitude of − 2.55 W/g. With additive phantom also exhibits two endothermic features, but with substantially reduced intensity: a primary peak at − 4.08 °C with a heat-flow magnitude of − 0.73 W/g, and a secondary peak at − 2.21 °C with a heat-flow magnitude of − 0.52 W/g.

Differential Scanning Calorimetry (DSC) was used to compare the melting behavior of phantoms with and without the embedded additive, with the goal of characterizing the distribution of freezable and bound water within the hydrogel matrix. Both samples, shown in Fig. 11, exhibited two overlapping endothermic features in the sub-zero region, consistent with multiple water states present in PVA-based cryogels.

The phantom without additive displayed a broad, high-magnitude endothermic event, with peak heat-flow values of approximately − 2.55 W/g near − 2.13 °C and − 2.26 W/g near − 3.82 °C. The large peak intensities indicate a substantial amount of freezable water, including both free water and freezable bound water. In contrast, the phantom containing the additive exhibited markedly reduced endothermic responses in the same temperature range, with peaks of − 0.73 W/g at − 4.08 °C and − 0.52 W/g at − 2.21 °C. The reduced peak magnitudes reflect a lower quantity of crystallizable water.

Because melting enthalpy is proportional to the amount of water that crystallizes during cooling, the diminished peak areas in the additive-containing phantom indicate that a larger fraction of water exists in a non-freezable, polymer-bound state. Both samples melted within the same temperature window, suggesting similar water populations, but the relative proportions differed substantially.

The DSC results demonstrate that the embedded additive strengthens water-matrix interactions, reducing the amount of free and freezable bound water while increasing the proportion of tightly bound water. This thermal behavior aligns with the hydration and electrical stability trends observed in earlier sections, providing molecular-level evidence that the additive enhances water retention and suppresses dehydration-driven variability in phantom performance.

## Conclusion

This study establishes a reproducible framework for enhancing hydration retention and electrical stability in PVA-based synthetic skin phantoms through the combined use of hydrophilic additives and controlled freeze-thaw processing. Across all evaluated treatments, Aloe Vera emerged as the most effective additive, providing substantial improvements in moisture preservation and impedance consistency relative to untreated controls. Embedded at an optimal concentration of approximately 0.75 g (5.5–6.5% w/w), Aloe Vera minimized mass loss, reduced impedance and phase-angle drift, and extended phantom electrical viability over multi-day testing. Concentration-dependent analysis revealed a clear stabilization window: moderate additive loading improved ionic conduction pathways and reduced signal variability, while higher concentrations (≥ 1.25 g) produced oversaturation, matrix softening, and erratic electrical behavior.

Freeze-thaw optimization showed that a single cycle provides the best balance between polymer crosslinking and structural integrity. Additional cycles (≥ 2) accelerated dehydration, increased mass loss, and amplified electrical instability, indicating that repeated cycling degrades rather than strengthens the PVA network. Differential Scanning Calorimetry (DSC) provided molecular-level validation of these findings. Aloe-Vera-treated phantoms exhibited depressed freezing onset temperatures and reduced enthalpy, confirming a decrease in freezable water and an increase in bound-water fractions. This thermal evidence aligns with electrical and hydration measurements, demonstrating that Aloe Vera stabilizes the phantom by altering internal water distribution and reducing water mobility.

Together, these results define a clear optimal configuration for extended phantom stability: ~0.75 g Aloe Vera embedded within the PVA matrix combined with a single freeze-thaw cycle. This formulation produced an 18-day-stable phantom, a substantial improvement over the ~4-day electrical lifespan of the additive-free control and longer than the stability typically reported for PVA-based phantoms. The optimized formulation maintained hydration retention, electrical fidelity, and structural robustness across biopotential-relevant frequency ranges, establishing a durable and reproducible platform for long-duration biosensor evaluation. Future work will investigate mechanical loading effects and evaluate commercial electrode performance on the optimized phantom to further advance its applicability in wearable biosensor testing.

## Supplementary Information

Below is the link to the electronic supplementary material.

Supplementary Material 1

## Data Availability

The datasets generated and/or analyzed during the current study are not publicly available due to the sensitive nature of the experimental files and proprietary phantom formulation details, but are available from the corresponding author upon reasonable request. The data that support the findings of this study are available from the corresponding author, [Krittika Goyal, Email: krgmet@rit.edu], upon reasonable request.

## List of symbols

ECG: Electrocardiography
EMG: Electromyography
EEG: Electroencephalography
Z: Impedance (Ω)
θ: Phase angle (°)
f: Frequency (Hz)
SC: Stratum corneum
PDMS: Polydimethylsiloxane
BaTiO_3_: Barium titanate
PVA: Polyvinyl alcohol (used in skin phantom formulation)
RH: Relative humidity (%)
RT: Room temperature
AU: Arbitrary units (Corneometer hydration measurement)
DI Water: Deionized water
Hydrophilic additives: Substances used to retain moisture (e.g., glycerin, trehalose, urea)
Three electrode configuration: Three-electrode system used for skin phantom impedance and phase angle measurements
w/w: Weight-to-weight ratio
${A}_{pore}$: Area of the pore
${A}_{bulk\:material}$: Area of the bulk material
Ø: Porosity
SD: Standard deviation
FTF: Freeze-thaw
$\varDelta\:log$: Log-shift impedance change from day 1
h: Hours
DSC: Differential scanning calorimetry
