# Supplementary material for: Hydration-stable PVA-based skin phantom for wearable biopotential sensor evaluation
Source: Sci Rep. 2026 Apr 28;16:19514. doi: 10.1038/s41598-026-49790-8 (PMC13287695; doi:10.1038/s41598-026-49790-8)
Supplement: Supplementary file 1 — Supplementary Material 1 [file 41598_2026_49790_MOESM1_ESM.docx]

**Supplementary Information**

**Hydration-Stable PVA-Based Skin Phantom for Wearable Biopotential Sensor Evaluation**

1. **Visual Documentation of Skin Phantom Structure and Processing Effects**


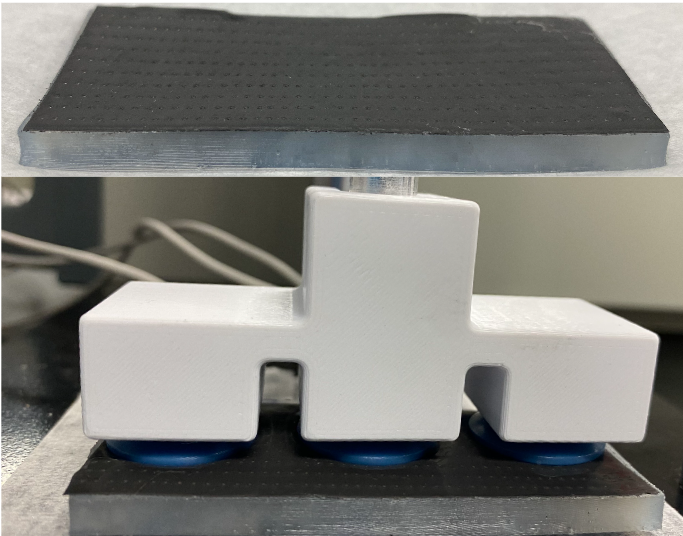


**Figure S1:** Photograph of the assembled two‑layer skin phantom used in electrical testing. The image shows the complete phantom consisting of a black PDMS upper layer bonded to a translucent PVA cryogel lower layer. The upper layer contains the patterned microperforations used for electrode contact, while the lower layer provides the hydrated conductive substrate characterized in this study. This image is provided to give visual context for the physical form of the phantoms tested.


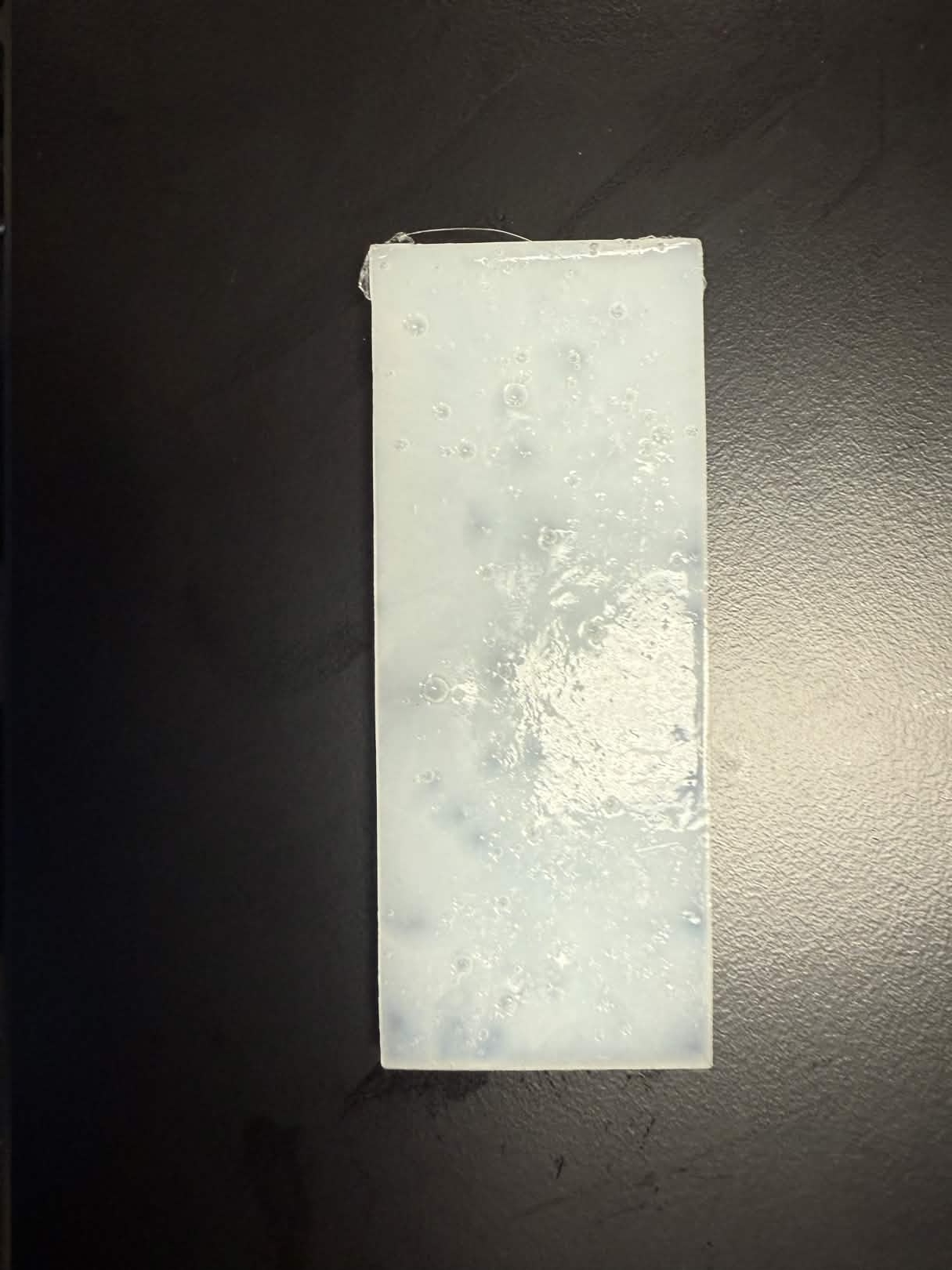


**Figure S2:** Photograph of the lower-layer PVA cryogel used in additive screening and freeze-thaw testing. The image shows the translucent PVA cryogel phantom in its standalone form, without the PDMS upper layer, enabling direct characterization of hydration retention, mass loss, and electrical stability. This figure provides visual context for the physical structure of the lower‑layer samples evaluated during additive and freeze-thaw optimization.

1. **Lower Layers with Aloe Vera Electrical Performance**


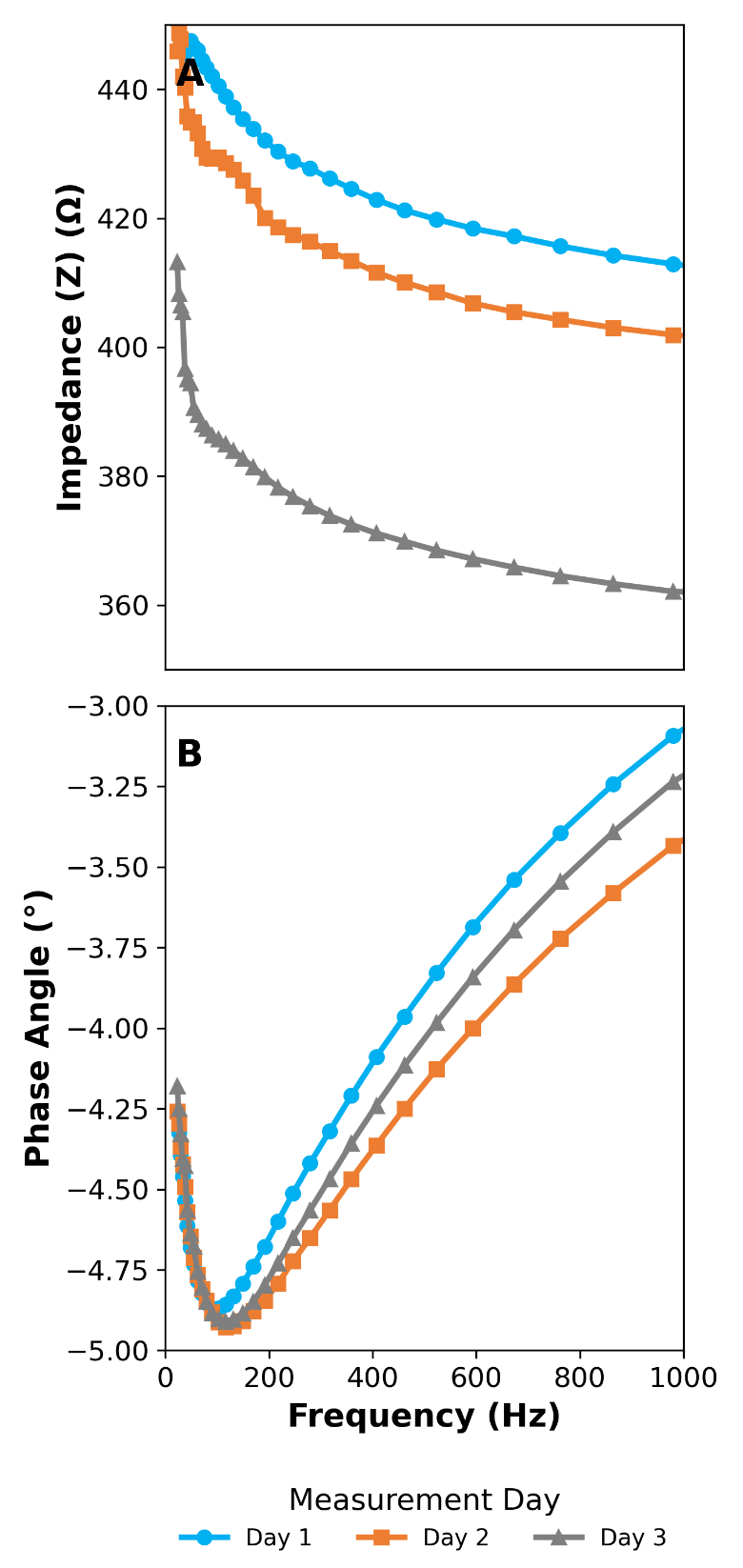


**Figure S3:** With Aloe Vera lower layer (A) impedance and (B) phase angle over frequency. Panel A depicts the impedance (Z) (Ω) versus frequency (Hz) for three measurement days: day 1 (highest impedance, decreasing from approximately 440 Ω to 415 Ω); day 2 (mid-range impedance, decreasing from approximately 435 Ω to 400 Ω); and day 3 (lowest impedance, decreasing from approximately 415 Ω to 360 Ω). Panel B depicts the phase angle (°) versus frequency (Hz); all three days follow a similar trend, dipping to a minimum of approximately -5.00° around 150 Hz, and then increasing, with day 1 showing the least negative phase angle and day 3 the most negative.

1. **Effect of Varying Aloe Vera Concentration and Porosity on Electrical Lifespan**

| **Aloe Vera Amount (g)** | **Aloe Vera Concentration (% w/w)** | **Porosity (%)** | **Days Before Electrical Death** | **Lifespan Difference With Additive** |
| --- | --- | --- | --- | --- |
| 0.2582 | 2.14 | 2.37 | 16 | 11 Days |
| 0.2582 | 2.00 | 1.54 | 13 | 8 Days |
| 0.3762 | 2.57 | 22.13 | 3 | 2 Days |
| 0.3762 | 2.79 | 20.00 | 3 | 2 Days |
| 0.3762 | 2.69 | 15.00 | 1 | -1 Day |
| 0.3762 | 2.61 | 11.56 | 2 | -1 Day |
| 0.50085 | 4.06 | 2.37 | 14 | 10 Days |
| 0.50085 | 4.18 | 1.54 | 14 | 9 Days |
| 0.7503 | 5.15 | 22.13 | 6 | 5 Days |
| 0.7503 | 8.11 | 20.00 | 6 | 5 Days |
| 0.7503 | 5.43 | 15.00 | 6 | 4 Days |
| 0.7503 | 6.21 | 11.56 | 10 | 7 Days |
| 0.7534 | 5.74 | 2.10 | 18 | 14 Days |
| 0.7534 | 6.70 | 1.24 | 18 | 13 Days |
| 1.0001 | 8.02 | 2.10 | 16 | 12 Days |
| 1.0001 | 7.79 | 1.24 | 16 | 11 Days |
| 1.2546 | 9.21 | 2.28 | 13 | 9 Days |
| 1.2546 | 10.24 | 1.51 | 12 | 7 Day |
| 1.5027 | 13.94 | 2.28 | 10 | 6 Days |
| 1.5027 | 15.17 | 1.51 | 9 | 4 Days |

**Table S1:** Effect of aloe vera additive concentration and porosity on phantom electrical lifespan. listed are the aloe vera mass and concentration, resulting porosity, number of days before electrical failure, and the corresponding lifespan difference relative to a no-additive control.

1. **Preliminary Freeze-Thaw Visual Testing**


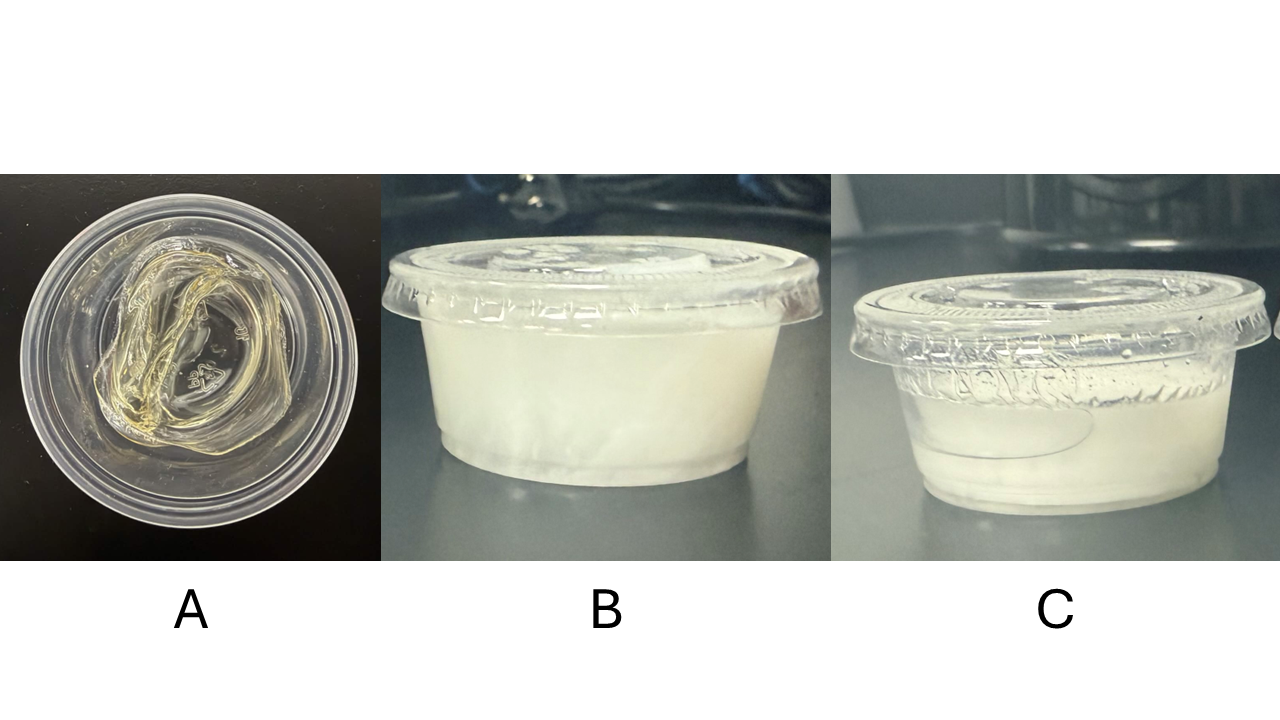


**Figure S4:** Visual comparison of lower‑layer phantom behavior under different processing conditions. Representative samples are shown after (left) curing at room temperature without freeze-thaw, (middle) exposure to a single freeze-thaw cycle, and (right) exposure to multiple freeze-thaw cycles. The room‑temperature sample cured into a typical transparent thermoplastic, whereas a single freeze-thaw cycle produced a structured, water‑retaining phantom suitable for electrical testing. In contrast, multiple freeze-thaw cycles led to visible water leakage and loss of structural integrity. These preliminary observations qualitatively support the selection of a single freeze-thaw cycle as an optimal processing condition.
